# Near‐Death Cells Cause Chemotherapy‐Induced Metastasis via ATF4‐Mediated NF‐*κ*B Signaling Activation

**DOI:** 10.1002/advs.202205835

**Published:** 2023-02-05

**Authors:** Chenchen Zhu, Pei Liu, Chuan‐Yuan Li, Yuli Zhang, Jiang Yin, Linlin Hou, Guopei Zheng, Xinjian Liu

**Affiliations:** ^1^ Department of Biochemistry School of Medicine Shenzhen Campus of Sun Yat‐sen University Shenzhen Guangdong 510275 China; ^2^ Department of Dermatology Duke University Medical Center Durham NC 27710 USA; ^3^ Cancer Research Institute and Cancer Hospital Guangzhou Medical University Guangzhou Guangdong 510180 China; ^4^ Bebetter Med Inc. Guangzhou Guangdong 510525 China

**Keywords:** ATF4, chemotherapy, metastasis, near‐death cells, nonclassical NF‐*κ*B pathway

## Abstract

Cytotoxic chemotherapy is a primary treatment modality for many patients with advanced cancer. Increasing preclinical and clinical observations indicate that chemotherapy can exacerbate tumor metastasis. However, the underlying mechanism remains unclear. Here, it is attempted to identify the mechanisms underlying chemotherapy‐induced cancer recurrence and metastasis. It is revealed that a small subpopulation of “near‐death cells” (NDCs) with compromised plasma membranes can reverse the death process to enhance survival and repopulation after exposure to lethal doses of cytotoxins. Moreover, these NDCs acquire enhanced tumorigenic and metastatic capabilities, but maintain chemosensitivity in multiple models. Mechanistically, cytotoxin exposure induces activating transcription factor 4 (ATF4)‐dependent nonclassical NF‐*κ*B signaling activation; ultimately, this results in nuclear translocation of p52 and RelB in NDCs. Deletion of *ATF4* in parental cancer cells significantly reduces colony formation and metastasis of NDCs, whereas overexpression of ATF4 activates the nonclassical NF‐*κ*B signaling pathway to promote chemotherapy‐induced metastasis of NDCs. Overall, these results provide novel mechanistic insights into the chemotherapy‐induced metastasis and indicate the pivotal role of NDCs in mediating tumor relapse after cytotoxic therapy. This study also suggests that targeting ATF4 may be an effective approach in improving the efficacy of chemotherapy.

## Graphical abstract

Lethal‐dose of chemotherapy induces tumor cell death. This work demonstrates that a small population of dying tumor cells can repair the compromised plasma membrane and reverse the process of cell death to survive. These surviving tumor cells acquire enhanced tumorigenic and metastatic capabilities by activating ATF4‐dependent nonclassical NF‐*κ*B signaling pathway, which provides a new mechanistic explanation for chemotherapy‐induced tumor metastasis.

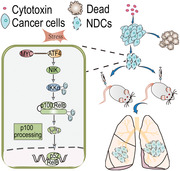

## 1 Introduction

Cytotoxic chemotherapy is a main therapeutic modality for patients with advanced cancer.^[^ ^1^ ^]^ Although many tumors can be reduced to undetectable levels by chemotherapy, tumors can reoccur at the original site or relapse as metastases to distant sites, which is responsible for about 9 out of 10 cancer deaths.^[^ ^2^ ^]^ A conventional explanation for tumor relapse is that chemotherapeutic agents exert a selection pressure that allows cancer cells resistant to these therapies to survive and thrive.^[^ ^3^, ^4^, ^5^ ^]^ However, emerging evidence has indicated that chemotherapeutic agents can promote tumor relapse in a noncancer cell‐extrinsic or cancer cell‐intrinsic manner. In a noncancer cell‐extrinsic manner, systemic administration of chemotherapeutic agents can generate a metastasis‐favorable tissue microenvironment and affect host endothelial, myeloid‐lineage, and immune‐related cells.^[^ ^6^, ^7^, ^8^ ^]^ For cancer cell‐intrinsic changes, chemotherapeutic agents can upregulate the expression of antiapoptotic genes and induce the migration and invasion of cancer cells. For example, cancer cells derived from 5‐fluorouracil (5‐FU)‐treated patients escape drug‐induced cell death by entering a stem cell‐like state, thereby becoming highly resistant to this drug.^[^ ^9^, ^10^ ^]^ Further, doxorubicin (DOX) can easily induce senescence in breast cancer cells, resulting in epithelial‐mesenchymal transition by activating the Notch signal pathway which promotes breast cancer metastasis.^[^ ^11^ ^]^ Cancer cells can also enter a reversible diapause‐like drug‐tolerant persister state to evade death and survive chemotherapy (5‐FU, leucovorin, and oxaliplatin) and targeted agents (osimertinib, dabrafenib, and lapatinib); these cells achieve this by regulating fatty acid metabolism under long‐term drug stress.^[^ ^12^, ^13^, ^14^ ^]^ Despite these advances, there is not a comprehensive understanding of chemotherapy‐induced cancer recurrence and metastasis.

Chemotherapeutic agents kill cancer cells via various cell death pathways, including apoptotic cell death with plasma membrane blebbing and nonapoptotic cell death with plasma membrane rupture.^[^ ^15^ ^]^ Furthermore, secondary necrosis of apoptotic cells, a previously believed nonregulated form of cell lysis that occurs after apoptosis, can be programmed and executed by plasma membrane pore formation, similar to that of pyroptosis.^[^ ^16^ ^]^ The plasma membrane is central for homeostatic maintenance in mammalian cells. The loss of plasma membrane integrity is considered the “point‐of‐no‐return” in cell death, according to the Nomenclature Committee on Cell Death (NCCD) morphological criteria.^[^ ^17^ ^]^ The prevailing view is that the process of plasma membrane integrity loss will definitively end cellular life. However, whether all cells that have lost membrane integrity after chemotherapy eventually die has not yet been experimentally investigated.

In this study, we carefully examined the survival of cancer cells exposed to a lethal dose of cytotoxins. Unexpectedly, we found that a small subpopulation of dying cells with compromised plasma membranes can survive and repopulate while maintaining chemosensitivity. The repopulating cancer cells acquired enhanced tumorigenic and metastatic capacities by inducing activating transcription factor 4 (ATF4)‐dependent nonclassical NF‐*κ*B signaling activation.

## 2 Results

### 2.1 Identification of Subpopulation of Near‐Death Cancer Cells

**Figure 1:**
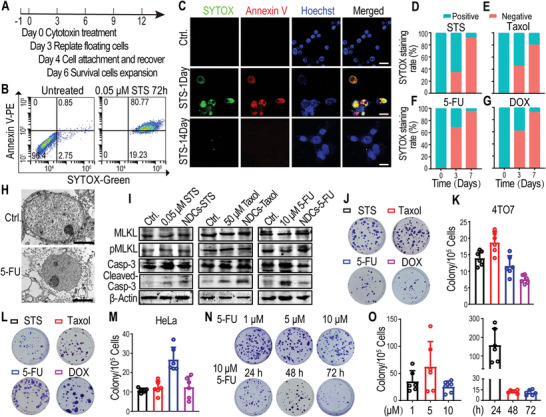
Identification of a subpopulation of near‐death cancer cells (NDCs) derived from cancer cell lines. A) Schematic representation of NDC induction using lethal concentrations of the chemotherapeutic drugs. B) Flow cytometry analyses of the plasma membrane integrity and apoptosis of floating cells (4TO7 cells treated with 0.05 × 10^−6^ m staurosporine [STS] for 72 h) using SYTOX‐Green/Annexin V‐PE dye. C) Confocal images of re‐adherent cells derived from floating cells (4TO7 cells treated with 0.05 × 10^−6^ m STS for 72 h) after the indicated recovery times by staining with Annexin V‐PE/Hoechst/SYTOX‐Green. SYTOX‐Green‐positive staining in the nucleus, representing the loss of NDC plasma membrane integrity. The scale bars represent 25 µm. D–G) The percentage of SYTOX‐Green‐positive adherent cells derived from floating 4TO7 cells after the indicated recovery times. The floating 4TO7 cells were collected after treatment with D) STS (0.05 × 10^−6^ m), E) Taxol (50 × 10^−6^ m), F) 5‐fluorouracil (5‐FU; 10 × 10^−6^ m), or G) doxorubicin (DOX; 1 × 10^−6^ m) for 72 h; cells were re‐plated to recover for the indicated times, then stained with SYTOX‐Green. The data represent the mean of three biological replicates. H) Transmission electron microscopy scanning of parental HeLa cancer cells (Ctrl.) and re‐adherent 5‐FU‐treated HeLa cells after 14 days of culture. The scale bars represent 1 µm. I) Western blot analysis of the expression of apoptosis (cleaved caspase‐3) and necroptosis (pMLKL) markers in cytotoxin‐treated cells (harvested 72 h after treatment) and cytotoxin‐derived 4TO7 NDCs. *β*‐Actin was used as the protein loading control. J–M) Representative J,L) crystal violet staining and K,M) numbers of colonies derived from 1 × 10^5^ cytotoxin‐induced floating J,K) 4TO7 cells and L,M) HeLa cells. N,O) Representative N) crystal violet staining (upper panel) and O) numbers (left panel) of repopulated colonies derived from 1 × 10^5^ floating 4TO7 cells that were treated with different concentrations of 5‐FU for 72 h. Representative N) crystal violet staining (lower panel) and O) numbers (right panel) of repopulated colonies derived from 1 × 10^5^ floating 4TO7 cells that were harvested at the indicated time after 10 × 10^−6^ m 5‐FU treatment. *n* = 6 biological replicates in (K, M, O).

To examine the fate of cytotoxin‐treated cancer cells, we treated 4TO7 cells with apoptotic cell death inducer staurosporine (STS) at dose of 0.05 × 10^−6^ m for 72 h (**Figure** **1**A). STS‐treated cells detached and floated in the culture medium (Figure S1A, Supporting Information). Flow cytometry analysis indicated that >80% of floating cells stained positive for Annexin V, an apoptotic marker for the loss of plasma membrane asymmetry (Figure 1B). Further, the plasma membrane integrity of floating cells was detected using SYTOX‐Green, a high affinity nucleic acid dye that is impermeant to live cells with intact plasma membrane, but quickly penetrates the compromised plasma membranes of dying/dead cells. Interestingly, all floating cells were observed to be positive for SYTOX‐Green staining (Figure 1B), indicating a loss of plasma membrane integrity of the floating cells.

To further investigate whether the floating cells were all dead after the loss of plasma membrane integrity, we re‐plated them; strikingly, we found a small subpopulation of cancer cells attached to the plate within 24 h following STS treatment (Figure 1A and Figure S1A, Supporting Information) or other chemotherapeutic agent treatment, such as paclitaxel (Taxol), 5‐FU, and DOX. SYTOX‐Green staining showed that the adherent cells derived from the floating cells possessed compromised plasma membranes, even after the reattachment (Figure 1C and Figure S1B, Supporting Information). The proportion of SYTOX‐Green‐positive adherent cells was reduced in a time‐dependent manner (Figure 1D–G and Figure S1C, Supporting Information), suggesting that the adhered membrane‐penetrating cancer cells may have strong self‐repairing abilities. However, upon comparing re‐adherent 5‐FU‐treated cells on day 14 to parental cancer cells using transmission electron microscopy (TEM), different sizes and morphological features on the outer leaflet of the plasma membrane and intensities of intercellular elements were observed (Figure 1H). Interestingly, some re‐adherent cells were still positive for the dying‐cell indicators, SYTOX‐Green and propidium iodide (PI), for 2–4 weeks after treatment, with gradual phosphatidylserine recovery in the plasma membrane to a relatively low level (Figure 1C and Figure S1D–G, Supporting Information). Surprisingly, although the re‐adherent SYTOX‐Green‐ or PI‐positive cancer cells with compromised plasma membranes were supposed to be dead according to the prevailing cell biology paradigm, we observed that these cells could, in fact, recover and proliferate (Figure 1C and Figure S1H, Supporting Information). We referred these surviving cells with compromised plasma membrane as “near‐death cells” (NDCs). These cytotoxin‐induced NDCs could proliferate despite caspase‐3 activation (Figure 1I and Figure S1I–K, Supporting Information) to form colonies after exposure to lethal dose of chemotherapeutic agents; this was observed across breast cancer, cervical cancer, ovarian cancer, and colorectal cancer cells (Figure 1J–M and Figure S1L, Supporting Information). The colony‐forming capability of 5‐FU‐induced NDCs was independent of the tested 5‐FU concentrations (Figure 1N,O). Furthermore, long‐term exposure to chemotherapeutic agents did not eradicate the colony‐forming ability of NDCs (Figure 1N,O and Figure S1M,N, Supporting Information). These results demonstrated that, following lethal doses of chemotherapeutic agents, a small subpopulation of cancer cells could survive and repopulate despite compromised plasma membranes and caspase‐3 activation.

### 2.2 Drug Sensitivity of NDCs

**Figure 2:**
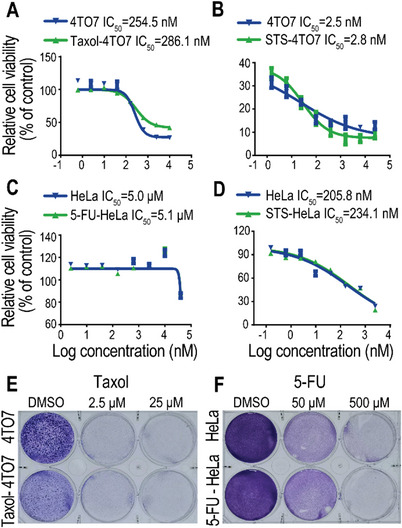
Drug sensitivity of NDCs. A) Taxol or B) STS sensitivity of parental 4TO7 cells, Taxol‐induced 4TO7 NDCs (30 days recovery after 50 × 10^−6^ m Taxol treatment for 72 h), and STS‐induced 4TO7 NDCs (30 days recovery after 0.05 × 10^−6^ m STS treatment for 72 h). *n* = 3 biological replicates. C) 5‐FU or D) STS sensitivity of parental HeLa cells, 5‐FU‐induced HeLa NDCs (30 days recovery after 20 × 10^−6^ m 5‐FU treatment for 72 h), and STS‐induced HeLa NDCs (30 days recovery after 0.1 × 10^−6^ m STS treatment for 72 h). *n* = 3 biological replicates. E) Representative crystal violet staining of parental 4TO7 cells and Taxol‐induced NDCs treated with 2.5 × 10^−6^ or 25 × 10^−6^ m Taxol for 48 h. F) Representative crystal violet staining of parental HeLa cells and 5‐FU‐induced NDCs treated with 50 × 10^−6^ or 500 × 10^−6^ m 5‐FU for 48 h.

The efficacy of cancer chemotherapy is often affected by the emergence of drug‐resistant cancer cells. To analyze whether the repopulation of NDCs was due to the presence of drug‐resistant cells, we carried out in vitro cytotoxicity tests. Half‐maximal inhibitory concentrations of NDCs derived from Taxol‐ and STS‐treated 4TO7 cells were observed to be similar to those of parental cancer cells treated with the same drugs (**Figure** **2**A,B). NDCs derived from cytotoxin‐treated HeLa cells also retained drug sensitivity comparable to that of the parental HeLa cells (Figure 2C,D). We further confirmed the drug sensitivity of cytotoxin‐induced NDCs using cell viability staining (Figure 2E,F). These results demonstrate that the survival and repopulation of near‐death cancer cells were not due to their resistance to chemotherapeutic agents.

### 2.3 Exacerbated Metastatic Capability of NDCs in Xenograft Model

**Figure 3:**
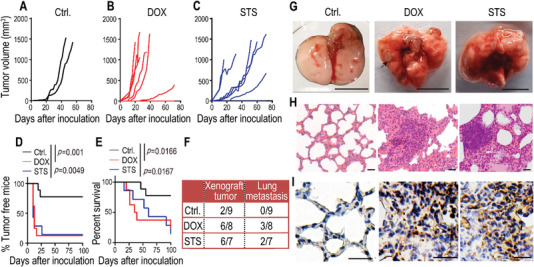
Exacerbated metastatic capability of NDCs in a xenograft model. A–C) 8 × 10^5^ luciferase‐labeled HeLa cells (HeLa‐Luc, Ctrl.) and DOX‐ or STS‐induced HeLa‐Luc NDCs were subcutaneously inoculated into female BALB/c‐nude mice. A) Xenograft tumors occurred in mice with HeLa‐Luc (*n* = 9), B) NDCs derived from DOX‐treated HeLa‐Luc (*n* = 8), and C) STS‐treated HeLa‐Luc (*n* = 7). D) Tumor‐free survival and E) overall survival of mice from the above experiments (A–C). *p* values were determined by a Log‐rank *t* test. F) The lung metastasis rates of HeLa‐Luc xenograft mice from the above experiments (A–C) on day 72 after inoculation. G) Gross photography showing apparent pulmonary metastases in xenograft mice with DOX‐ and STS‐induced HeLa‐Luc NDCs. The scale bars represent 1 cm. The black arrow indicates the site of the tumor nodule. H) H&E staining and I) IHC staining (anti‐luciferase antibody) of lung sections that show obvious metastatic masses in DOX‐ and STS‐induced HeLa‐Luc NDCs. The scale bars represent 50 µm for (H), and 20 µm for (I).

To evaluate the tumorigenicity of NDCs, we used a weak tumorigenicity cancer cell line, HeLa,^[^ ^18^ ^]^ for the xenograft model. Luciferase‐labeled HeLa cells (HeLa‐Luc) were treated with 0.5 × 10^−6^ m DOX or 0.1 × 10^−6^ m STS for 72 h. De‐attached floating HeLa‐Luc cells were re‐plated and cultured for ≈30 days; then, the repopulated cells were subcutaneously inoculated into BALB/c‐nude mice. The percentage of mice that developed subcutaneous tumors from NDCs derived from DOX‐ or STS‐treated HeLa‐Luc cells was determined to be much higher than that from the parental HeLa‐Luc cells (**Figure** **3**A–C). These results indicate that NDCs exhibit significantly enhanced tumorigenicity. The time of occurrence of palpable tumors was much earlier in mice inoculated with DOX‐ or STS‐induced HeLa‐Luc‐derived NDCs (Figure 3D); further, mice bearing NDC‐derived tumors possessed significantly shorter survival times (Figure 3E) than those inoculated with parental HeLa‐Luc cells. Additionally, we observed distant metastatic tumors in the lungs of DOX‐ and STS‐treated NDCs groups, but no obvious tumor nodules in the lungs of parental HeLa‐Luc xenograft mice 72 days after xenograft inoculation (Figure 3F,G). Representative hematoxylin and eosin (H&E, Figure 3H) and immunohistochemical (IHC) staining with luciferase antibodies (Figure 3I) confirmed the presence of a metastatic lung tumor mass in the NDCs groups. These data demonstrate that chemotherapy‐induced NDCs acquire enhanced tumorigenic and metastatic capabilities.

### 2.4 Enhanced Metastatic Capability of NDCs in Intravenous Model

**Figure 4:**
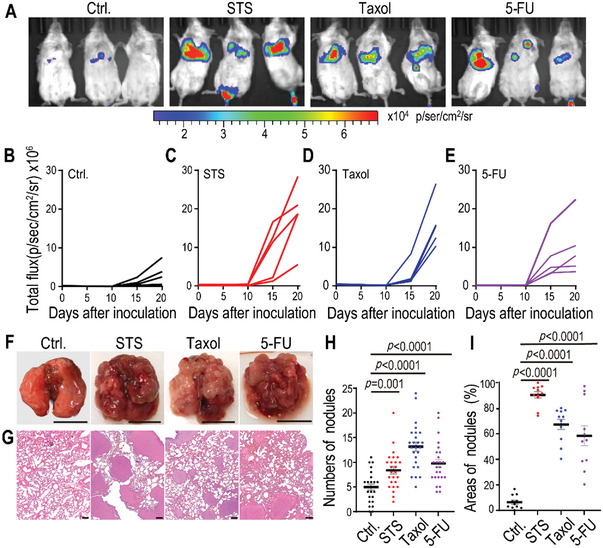
Enhanced metastatic capability of NDCs in an intravenous model. A–E) 5 × 10^5^ luciferase‐labeled 4TO7 cells (4TO7‐Luc, Ctrl.) and STS‐, Taxol‐, or 5‐FU‐induced 4TO7‐Luc NDCs were intravenously inoculated into female BALB/c mice. A) Bioluminescent representative images showing lung metastatic tumors in mice on day 20 after tail vein injection. The dynamic bioluminescence signals of lung metastatic tumors in mice with B) 4TO7‐Luc and C) STS‐, D) Taxol‐, or E) 5‐FU‐induced 4TO7‐Luc NDCs. *n* = 5 mice per group. F) Representative gross photography showing apparent pulmonary metastases in the lungs of mice at the end of the experiments (day 20). The scale bars represent 1 cm. G) H&E staining of lung sections showing obvious metastatic masses in STS‐, Taxol‐, and 5‐FU‐induced 4TO7‐Luc NDCs groups. The scale bars represent 200 µm. H) The number of metastatic tumor burden in the lung sections (*n* = 25 per group). I) The percentage of tumor area covering lung sections (*n* = 11 per group). Data are presented as mean ± standard error of the mean (SEM) in (H) and (I). *p* values were determined using Student's *t*‐test in (H) and (I).

To further evaluate the metastatic efficacy of cytotoxin‐induced NDCs, we used mouse breast cancer cells 4TO7, which are highly tumorigenic but very rarely metastatic cell line.^[^ ^19^ ^]^ Luciferase‐labeled 4TO7 cells (4TO7‐Luc) were treated with 0.05 × 10^−6^ m STS, 50 × 10^−6^ m Taxol, or 10 × 10^−6^ m 5‐FU for 72 h; the floating cells were observed to exhibit a loss in plasma membrane integrity. The floating 4TO7‐Luc cells were re‐plated and cultured for ≈25 days, and then the repopulated cells were injected into BALB/c female mice via the tail vein. Tumor dynamic metastasis capability was evaluated using in vivo imaging system (IVIS)‐bioluminescence imaging. 20 days after tail vein injection, NDC‐inoculated mice cells showed significantly enhanced luminescent signal intensity compared to those from mice with untreated 4TO7‐Luc cells (**Figure** **4**A). Chemotherapy‐induced NDCs colonized earlier and expanded faster in the lungs than parental 4TO7‐Luc cells (Figure 4B–E). Gross images also showed apparent pulmonary metastases in mice injected with cytotoxin‐induced NDCs. In contrast, few metastatic tumors were detected in the mice injected with untreated 4TO7‐Luc cells (Figure 4F). Further, histological analyses confirmed the increased number of pulmonary metastatic nodules and areas in mice injected with cytotoxin‐induced NDCs (Figure 4G–I). These results demonstrated that rarely metastatic 4TO7 breast cancer cells acquired enhanced metastatic capability after exposure to chemotherapeutic agents.

To further evaluate the lung colonization ability of floating cells without recovery, 4TO7‐Luc cells were treated with 0.05 × 10^−6^ m STS or 50 × 10^−6^ m Taxol for 5 days; then, these floating cells were collected and injected into BALB/c mice via tail vein. IVIS bioluminescence imaging indicated that STS or Taxol treatment could exacerbate the metastasis of 4TO7‐Luc cancer cells (Figure S2A,B, Supporting Information). Additionally, gross tissue analysis confirmed obvious lung and kidney metastases in mice inoculated with floating NDCs without recovery, when compared with the untreated 4TO7‐Luc cells group (Figure S2C,D, Supporting Information).

### 2.5 ATF4 Promotes NDCs‐Derived Lung Metastasis and is Consistent with Human Cancers

**Figure 5:**
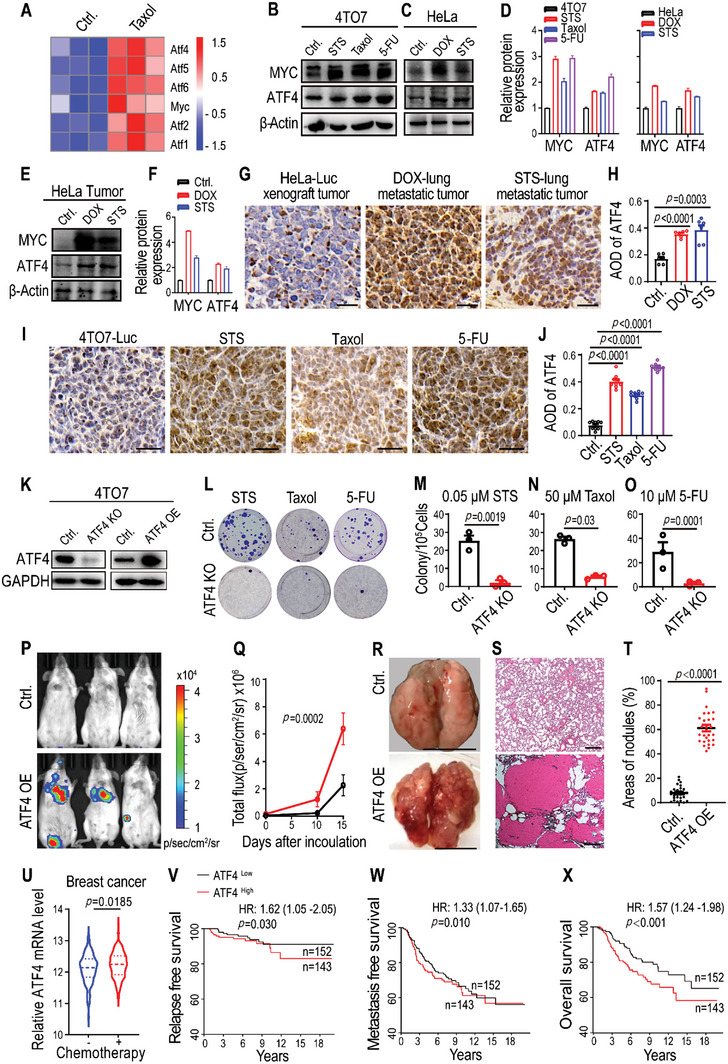
ATF4 promotes NDCs‐derived lung metastasis and is consistent with human cancers. A) Heatmap of transcription factor‐related genes in 4TO7 cells (Ctrl.) and Taxol‐induced 4TO7 NDCs (25 days recovery after 50 × 10^−6^ m Taxol treatment for 72 h). *n* = 3 biological replicates. B,C) Western blot images and D) quantification of MYC and ATF4 expression in control cells (Ctrl.), B) STS‐, Taxol‐, and 5‐FU‐induced 4TO7 NDCs, and C) DOX‐ and STS‐induced HeLa NDCs. E) Western blot images and F) quantification of MYC and ATF4 expression in tumor tissues of HeLa‐Luc control and DOX‐ and STS‐induced NDCs. G,I) IHC staining and H,J) quantification of ATF4 in tumors derived from cytotoxin‐induced G) HeLa NDCs and I) 4TO7 NDCs. The scale bars represent 50 µm. Data were taken from six random photography fields for (G) and nine random photography fields for (I); data are presented as mean ± SEM. K) ATF4 expression in 4TO7 cells with an ATF4 knockout (ATF4 KO) (left panel), and ATF4 over‐expression ATF4 (ATF4 OE) (right panel). GAPDH was used as a protein loading control. L) Representative crystal violet staining and the numbers of repopulated colonies from 1 × 10^5^ floating 4TO7 or 4TO7‐ATF4 KO cells that were collected and re‐plated after M) 0.05 × 10^−6^ m STS, N) 50 × 10^−6^ m Taxol, or O) 10 × 10^−6^ m 5‐FU treatment for 72 h. Data are presented as mean ± SEM. *n* = 3 biological replicates. P) Representative bioluminescent images taken 15 days after intravenous inoculation of BALB/c mice with 5 × 10^5^ 4TO7‐Luc cells (Ctrl.) and ATF4‐overexpression 4TO7‐Luc cells. Q) Quantitative analysis of the bioluminescence intensity from (P). Data are presented as mean ± SEM. *n* = 5 mice per group. *p* values were determined by a two‐way ANOVA. R) Representative gross photography showing apparent pulmonary metastases in the lungs of mice that received intravenous inoculation with ATF4‐overexpressing 4TO7‐Luc cells at the end of the experiments (day 20). The scale bars represent 1 cm. S) H&E staining showing obvious tumor masses in lung sections of mice that received intravenous inoculation with ATF4‐overexpressing 4TO7‐Luc cells. The scale bars represent 200 µm. T) The percentage of tumor area covering lung sections in (S). Data are presented as mean ± SEM. *n* = 27 photography fields per group. U) Chemotherapeutic agents induce ATF4 expression in human breast cancer. The *ATF4* gene expression data from breast cancer patients with (*n* = 198) or without (*n* = 103) chemotherapy were taken from GSE25065 or GSE22093. Data are presented as mean ± SEM. *p* values were determined using a Student's *t*‐test for (H), (J), (M), (N), (O), (T), and (U). V–X) High ATF4 expression correlates with worsened V) relapse‐free survival, W) metastasis‐free survival, and X) overall survival in human breast cancers. Patient data from microarray datasets (Netherlands Cancer Institute, NKI) were arbitrarily classified into ATF4‐high and ATF4‐low groups. The online tool PROGgene V2 was used, and the Kaplan–Meier curves of survival are shown. For the log‐rank test, *p* values and hazard ratios (HR) are indicated.

To investigate the NDCs‐mediated mechanism of chemotherapy‐induced metastasis, we carried out RNA sequencing (RNA‐seq) of NDCs derived from cytotoxin‐treated 4TO7 and parental cells. We identified 1835 genes that were significantly increased in NDCs derived from Taxol‐treated 4TO7 cells (Figure S3A, Supporting Information); further, Gene Ontology (GO) term enrichment of transcripts determined genetic associations with tumor proliferation and metastasis, such as genes involved in positive regulation of cell migration, cell proliferation, TGF‐*β* signaling, and MAPKK signaling (Figure S3B, Supporting Information).^[^ ^20^, ^21^ ^]^ Further analyses indicated that a panel of 23 tumor metastasis‐related genes (Figure S3C, Supporting Information) was highly expressed in the Taxol‐induced NDCs. This was consistent with the gene panel of metastasis in breast cancer patients with worse overall survival.^[^ ^22^ ^]^ Additionally, functional enrichment analyses revealed that the top 10 enriched Kyoto Encyclopedia of Genes and Genomes (KEGG) pathways were associated with transcription activator activity, thereby suggesting a key role for transcription activators in chemotherapy‐induced metastasis of NDCs (Figure S3D, Supporting Information). We further identified that numerous genes in the activating transcription factor/cyclic AMP response element binding (ATF/CREB) family and the oncogene *MYC* were highly expressed in NDCs (**Figure** **5**A); this was determined to be consistent with the public microarray data from Taxol and eribulin (microtubule inhibitor)‐treated cancer cells (Figure S3E, Supporting Information). ATF4, a member of the ATF/CREB family of transcription factors, is involved in supporting the survival of cancer cells undergoing external pressure, such as MYC‐induced stress.^[^ ^23^, ^24^ ^]^ Our data indicated that *ATF4* mRNA expression levels were elevated in cytotoxin‐induced 4TO7 NDCs (Figure S3F, Supporting Information) and primary or metastatic tumor tissues derived from cytotoxin‐induced HeLa‐Luc NDCs (Figure S3G,H, Supporting Information). Western blot analysis revealed that MYC and ATF4 were highly expressed in NDCs derived from cytotoxin‐treated cancer cells (Figure 5B–D) and tumors derived from cytotoxin‐treated HeLa‐Luc NDCs (Figure 5E,F). Finally, IHC staining analyses further confirmed the higher ATF4 expression in metastatic tumors from cytotoxin‐treated 4TO7‐Luc and HeLa‐Luc NDCs (Figure 5G–J).

To investigate the potential role of ATF4 in tumor growth, we first generated ATF4 knockout (ATF4 KO) and over‐expressing (ATF4 OE) 4TO7 and HeLa cell lines (Figure 5K and Figure S3I, Supporting Information). ATF4 knockout or parental cells were treated with STS, Taxol, or 5‐FU for 72 h. Then, the floating cancer cells were collected and re‐plated for colony formation. Our results indicated that an ATF4 knockout significantly reduces the number of NDCs colonies from cytotoxin‐treated ATF4 KO 4TO7 and HeLa cells (Figure 5L–O and Figure S3J–M, Supporting Information). Overexpression of ATF4 significantly exacerbated the metastatic capability of rarely metastatic 4TO7‐Luc cancer cells in BALB/c mice (Figure 5P–T).

We investigated whether ATF4 expression is relevant in human cancers. Public microarray datasets analysis showed that ATF4 expression was higher in breast tumors treated with chemotherapy than in those not treated with chemotherapy (Figure 5U). High ATF4 expression correlated with tumor relapse, metastasis, and reduced overall survival of patients with breast cancer (Figure 5V–X), adenoid cystic carcinoma (ACC), osteosarcoma, glioma, and kidney renal clear cell carcinoma (KIRC; Figure S3N–Q, Supporting Information). The worse outcome in patients with high ATF4 expression was consistent with our mouse tumor data, demonstrating that wild‐type tumors had stronger tumorigenicity and metastatic capability than their ATF4 knockout counterparts.

### 2.6 NDCs Promote Lung Metastasis by Enhancing the ATF4‐Mediated Noncanonical NF‐*κ*B Signaling Pathway

**Figure 6:**
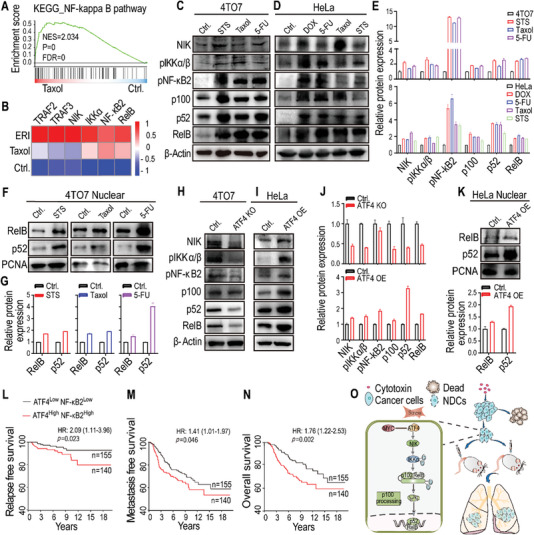
Cytotoxin‐induced NDCs promote lung metastasis by enhancing the noncanonical NF‐*κ*B signaling pathway. A) Gene set enrichment analysis (GSEA) showing the enrichment of the NF‐*κ*B signaling pathway in Taxol‐induced 4TO7 NDCs. B) Heatmap analyses of 27 breast cancer cell lines showing high expression of noncanonical NF‐*κ*B pathway genes in Taxol and Eribulin (ERI)‐treated breast cancer cells. C,D) Western blot images and E) quantification showing the activation of noncanonical NF‐*κ*B signals (NIK, phosphor‐IKK*α*/*β*, phosphor‐NF‐*κ*B2, p100, p52, and RelB) in cytotoxin‐induced C) 4TO7 NDCs (upper panel) and D) HeLa NDCs (lower panel). F) Western blot images and G) quantification of cellular fragments showing high expression of RelB and p52 in the nucleus of cytotoxin‐induced 4TO7 NDCs. H) Downregulation of noncanonical NF‐*κ*B signals in ATF4 KO 4TO7 cells. I) Upregulation of noncanonical NF‐*κ*B signals in ATF4‐overexpressing HeLa cells (ATF4 OE). J) Quantification of (H) and (I). *β*‐Actin was used as the protein loading control for (C), (D), (H), and (I). K) Increased RelB and p52 expression in the nucleus of ATF4‐overexpressing HeLa cells (ATF4 OE). Proliferating cell nuclear antigen (PCNA) was used as a loading control of the nuclear extracts for (F) and (K). L–N) High co‐expression of ATF4/NF‐*κ*B2 correlates with worsened L) relapse‐free survival, M) metastasis‐free survival, and N) overall survival in human breast cancers. Patient data from microarray datasets (Netherlands Cancer Institute, NKI) were arbitrarily classified into ATF4‐high/NF‐*κ*B2‐high and ATF4‐low/NF‐*κ*B2‐low groups. The online tool PROGgene V2 was used, and the Kaplan–Meier curves of survival are shown. For the log‐rank test, *p* values and hazard ratios (HR) are indicated. O) Diagrammatical image illustrating that, in multiple mouse models, lethal cytotoxin‐treated cancer cells can enter a state of near‐death with compromised plasma membrane, then reverse the death process to repopulate; these cells gain enhanced malignancy and exacerbated metastatic capability via ATF4‐mediated nonclassical NF‐*κ*B signaling pathway.

To elucidate how ATF4 promotes lung metastasis, we performed gene set enrichment analysis (GSEA) of the RNA‐seq data; consequently, the nuclear factor‐*κ*B (NF‐*κ*B) signaling pathway was determined to be significantly upregulated in cytotoxin‐induced 4TO7 NDCs (**Figure** **6**A and Figure S4A,B, Supporting Information), which is consistent with the public microarray data from chemotherapeutic agent‐treated cancer cells (Figure 6B). Activated NF‐*κ*B transcription factors are strongly associated with tumorigenesis, with properties such as enhance cancer cell proliferation, suppression of apoptosis, and increased potential for metastatic inflammation. We investigated dynamic changes in NF‐*κ*B signaling pathway markers, including NIK, IKK*α*/*β*, NF‐*κ*B2 (p100/p52), and RelB. Western blotting results indicated that the levels of phosphorylated IKK*α*/*β* (pIKK*α*/*β*) and phosphorylated NF‐*κ*B2 (pNF‐*κ*B2), which are the key kinases of the activated noncanonical NF‐*κ*B pathway, were upregulated in cytotoxin‐induced NDCs cancer cells, alongside other components, such as NIK, p100, p52, and RelB (Figure 6C–E and Figure S4C,D, Supporting Information). NF‐*κ*B inducing kinase (NIK) activates IKK*α*, which leads to the phosphorylation and processing of NF‐*κ*B2 (p100) to generate p52, thereby resulting in the formation of RelB/p52 complexes that activate the noncanonical NF‐*κ*B signaling pathway.^[^ ^25^, ^26^ ^]^ We observed increased nuclear translocation of RelB and p52 in cytotoxin‐induced NDCs (Figure 6F,G). Furthermore, quantitative reverse transcriptase polymerase chain reaction (qRT‐PCR) analyses confirmed the enhanced expression of *RelB* and *NF‐κB2* in cytotoxin‐induced NDC‐cancer cells and NDC‐derived metastatic tumor tissues (Figure S4E–G, Supporting Information).

The ATF/CREB family of proteins interacts with multiple domains of CREB‐binding protein (CBP), which are functionally essential for other transcription factors, such as NF‐*κ*B, TP53, and STATs.^[^ ^27^, ^28^ ^]^ Knockout of ATF4 significantly decreased the expression of the noncanonical NF‐*κ*B pathway components in cancer cells (Figure 6H, upper panel of Figure 6J), whereas overexpression of ATF4 increased the corresponding expression of these noncanonical NF‐*κ*B pathway components (Figure 6I, lower panel of Figure 6J), with nuclear translocation of RelB and p52 (Figure 6K). A two‐gene prognosis risk model revealed that, compared to patients with lower co‐expression, those with higher co‐expression of ATF4 and NF‐*κ*B2 showed a significantly stronger correlation with worsened relapse, metastasis, and reduced overall survival in patients with breast cancer (Figure 6L–N), ACC, osteosarcoma, glioma, and KIRC (Figure S4H–K, Supporting Information). Overall, our findings demonstrate that cytotoxin‐induced NDCs promote lung metastasis by enhancing the ATF4‐mediated noncanonical NF‐*κ*B signaling pathway.

## 3 Discussion

Here, we revealed that, after exposure to lethal doses of chemotherapy, a subpopulation of near‐death cancer cells with compromised plasma membranes could recover and repopulate. These NDCs maintain chemosensitivity as parental cancer cells and gain malignant properties; in particular, these NDCs demonstrate enhanced metastatic capacities through ATF4‐dependent nonclassical NF‐*κ*B signaling activation.

The plasma membrane is central to homeostatic maintenance of mammalian cells. Excessive mechanical or biochemical stresses disrupt plasma membrane integrity and, therefore, pose an immediate threat to cell survival. For example, a transient disruption in membrane integrity results in abruption of calcium influx,^[^ ^29^ ^]^ an immediate danger signal that triggers cellular degenerative biochemical and structural events to initiate cell death cascades.^[^ ^30^ ^]^ Cytotoxic chemotherapy, such as 5‐FU, paclitaxel, and DOX treatment, disrupts the plasma membrane in later cytotoxic processes; this eventually leads to cell death through multiple death modalities, including secondary necrosis and a progressive loss of plasma membrane integrity of apoptotic cells.^[^ ^31^ ^]^ Dying cells with loss of plasma membrane integrity and caspase activation would definitively end cellular life according to the NCCD criteria.^[^ ^17^ ^]^ However, we experimentally proved that even after exposure to lethal doses of cytotoxins, a small subpopulation of dying cancer cells with compromised plasma membranes could reverse the cell death process to survive and repopulate. Our findings demonstrate that plasma membrane changes during the cell death process are evidently more complicated than previously expected. Furthermore, these repopulating cancer cells are characterized by elevated cleaved caspase‐3 expression. This is consistent with our previous studies demonstrating that stress‐induced sub‐lethal caspase‐3 activation promotes carcinogenesis and sustains tumorigenicity and “stemness” of cancer cells.^[^ ^32^, ^33^, ^34^ ^]^

Modern chemotherapy modalities, including enhanced chemodynamic therapy,^[^ ^35^ ^]^ offer long‐term potential benefits to many patients with advanced cancers. However, accumulating preclinical and clinical evidence^[^ ^36^, ^37^ ^]^ indicates the unexpected involvement of standard chemotherapy operations in enhancing tumor‐host reactive responses, thereby inadvertently supporting the survival and dissemination of tumor cell subpopulations. This paradoxical effect of chemotherapy results in local tumor relapse and distant metastasis by affecting the evolution of the primary tumor, circulation, and distant organs.^[^ ^38^, ^39^ ^]^ Chemotherapy‐induced dying cancer cells release adenosine triphosphate,^[^ ^40^ ^]^ or other microenvironmental stimuli, to directly activate cancer stem cell self‐renewal pathways and induce epithelial‐mesenchymal transition, which provides cancer cells with enhanced disseminating properties, cancer stem cell features, and chemo‐resistance.^[^ ^41^, ^42^, ^43^, ^44^, ^45^ ^]^ Furthermore, the rapid elimination of dying cancer cells in the microenvironment can trigger immunologically silent conditions to facilitate tumor immune escape and progression.^[^ ^46^ ^]^ In this study, we found that the survival of NDCs after a lethal dose of chemotherapy enhanced malignancy and metastatic capability toward distant organs. This paradoxical pro‐metastatic effect of chemotherapy‐derived NDCs does not rely on long‐term exposure; however, it occurs quickly after exposure to chemotherapy. Ultimately, these findings provide new insight in understanding the connection between chemotherapy and tumor metastasis.

ATF4 is a member of the activating transcription factor family, and its expression is increased in response to diverse microenvironmental stresses, including starvation, endoplasmic reticulum stress damage, and exposure to toxic factors.^[^ ^47^, ^48^ ^]^ ATF4 expression is elevated in tumors, especially in hypoxic and nutrient‐deprived regions, where it promotes metabolic homeostasis and cancer cell survival by translocating into the nucleus and transcriptionally regulating several genes required for protein synthesis and redox balance.^[^ ^49^, ^50^ ^]^ By mediating antioxidant response activation, ATF4 enables cancer cells to survive and migrate to secondary sites during tumor metastasis.^[^ ^51^ ^]^ A previous study demonstrated that upregulation of ATF4 correlates with resistance to chemotherapeutic agents, including cisplatin, DOX, etoposide, and vincristine.^[^ ^52^ ^]^ In our study, we further demonstrated that higher ATF4 expression in tumors is associated with worsened tumor relapse‐free survival, metastasis‐free survival, and overall survival of cancer patients. Comparative analysis of gene expression sets in chemical‐treated ATF4 deficient and wild‐type mouse embryonic fibroblasts demonstrated ATF4‐dependent modulation of canonical and noncanonical NF‐*κ*B signaling, including modulation of RelB, Traf3, and Traf2.^[^ ^53^ ^]^ Our finding that ATF4 is involved in NDC induction following chemotherapy is consistent with the ability of ATF4 to promote tumor progression and metastasis.^[^ ^51^, ^54^ ^]^ In chemotherapy‐treated stress‐induced NDCs, ATF4‐dependent nuclear translocation of the noncanonical NF‐*κ*B signaling factors RelB and p52 promotes tumor metastasis (Figure 6O); this is consistent with the understanding that chromosomal instability drives metastasis through nuclear localization of the noncanonical NF‐*κ*B p52/RelB complex.^[^ ^55^ ^]^ Nuclear immunostaining of NF‐*κ*B2 and RelB in tumor samples from a large cohort of breast cancer patients further demonstrated that noncanonical NF‐*κ*B pathway activation in these tumors can be used to predict poor survival in breast cancer patients.^[^ ^56^ ^]^ When considering ATF4 and NF‐*κ*B2, high co‐expression of these factor in tumors correlates with worsened outcomes of cancer patients compared to those with low expression (Figure 6L,M). Overall, these finding suggest that ATF4 inhibition is a promising target for diminishing tumor growth and metastasis.

## 4 Experimental Section

### Cell Culture

In the present study, a variety of cancer cell lines were used. Among these were the mouse breast cancer cell line 4TO7, and the human cancer cell lines HeLa (cervical cancer), COV‐504 (ovarian carcinoma), A2780 (ovarian carcinoma), and HCT116 (colorectal carcinoma). Additional details are provided in the Methods and Table S1 in the Supporting Information.

### Generation of NDCs

The corresponding treatments used for cancer cells in the current study were as follows: 4TO7 cells were treated with STS (0.05 × 10^−6^ m), Taxol (50 × 10^−6^ m), 5‐FU (10 × 10^−6^ m), or DOX (1 × 10^−6^ m); HeLa cells were treated with STS (0.1 × 10^−6^ m), Taxol (3 × 10^−6^ m), 5‐FU (20 × 10^−6^ m), or DOX (0.5 × 10^−6^ m); COV‐504 and A2780 cells were treated with STS (0.2 × 10^−6^ m); and HCT116 cells were treated with STS (0.05 × 10^−6^ m). Cancer cells were treated with a lethal dose of drugs to induce cell death, and floating cells were collected and cultured in fresh medium. Then, 24 h after plating, the cells were washed with phosphate‐buffered saline (PBS) and treated with the fluorescent nucleic acid dye SYTOX‐Green at a final dose of (250 × 10^−9^ m) for 15 min. After dye removal, cells were cultured and observed. Cells that emitted green fluorescence and formed clones were considered as NDCs.

### Fluorescent Dye Labeling Assay

After cytotoxin treatment, the floating cells were directly plated and labeled with annexin V‐PE, Hoechst33258 (1 × 10^−6^ m), and SYTOX‐Green (250 × 10^−9^ m) for flow cytometry or microscopic analyses.

### Colony Formation Assay

After cytotoxin treatment, floating cells were counted and seeded in 6‐well plates for colony formation. After culturing for 20 days, colonies were stained with 0.5% crystal violet. The number of colonies was then counted with a diameter over five pixels in the photographs using Image J software (https://ij.imjoy.io/).

### TEM

The morphology of the NDCs after 5‐FU (10 × 10^−6^ m) treatment was examined using a TEM (JEM1200‐EX, JEOL). The samples were collected on day 14 after re‐plating the floating HeLa cells; then cells were washed with PBS three times, and fixed with 2.5% glutaraldehyde for observation. Parental HeLa cells were used as the controls.

### Cell Apoptosis and Drug Sensitivity Analysis

For apoptosis analysis, Annexin V‐FITC/PI staining was performed according to the manufacturer's instructions (Annexin V‐FITC Apoptosis detection kit, MultiSciences, Cat#AP101). The samples were analyzed using a CytoFLEX Flow Cytometer (Beckman Coulter, Inc.). The 50% inhibitory dose (IC_50_) was defined as the drug concentration required to reduce cell proliferation to 50% of the untreated control. The IC_50_ values were calculated using GraphPad software (version 9); the log (inhibitor) versus response‐variable slope (four parameters) equation under a nonlinear regression dialog was used.

### Mouse Models

6‐ to 8 weeks old female BALB/c and BALB/c nude mice were obtained from the Sun Yat‐sen University Animal Facility. Mice were housed in an environmentally controlled room (temperature 23 ± 2 °C, relative humidity 30–70%) under specific pathogen‐free (SPF) conditions, fed irradiated laboratory rodent chow, and provided sterile water by the facility staff. The animal experimental procedures in this study were approved by the Sun Yat‐sen University Institutional Animal Use and Care Committee (SYSU‐IACUC‐2019‐B690).

HeLa‐Luc cells (8 × 10^5^) or HeLa‐Luc cell‐derived NDCs were injected subcutaneously into the flanks of BALB/c‐nude mice. Tumor size was measured with a caliper and calculated using the following formula: volume = (length)(width)^2^/2. The endpoint was defined as the time when a progressively growing tumor reached 15 mm in the longest dimension. Tumor samples were collected at the indicated time points and processed for bioanalysis. The tumor that reached the endpoint was removed and its growth was observed after resection.

5 × 10^5^ 4TO7‐Luc cells and NDCs derived from cytotoxin‐treated 4TO7‐Luc cells were intravenously injected into the tail veins of BALB/c mice. Mice were anesthetized with isoflurane (RWD, Cat#R510‐22‐10) and injected (intraperitoneally) with 150 mg kg^−1^ D‐luciferin potassium salt (Beyotime, Cat#ST196) at dose of (150 mg kg^−1^) on days 0, 10, 15, and 20 after tumor cell injection. 4TO7‐Luc lung metastases were assessed by in vivo bioluminescence imaging using IVIS Spectrum. The integrated light intensity, measured by single‐photon counting with 10 min exposure, was used to quantify the amount of light emitted by the 4TO7‐Luc cells. A low‐intensity visible‐light image was created for the overlay images.

### qRT‐PCR Analysis

Total RNA was extracted using TRIzol reagent (Invitrogen, Cat#44894), according to the manufacturer's protocol. Then, cDNA synthesis was performed using random hexamer primers and Superscript II Reverse Transcriptase (Invitrogen, Cat#18064‐014). qRT‐PCR was performed using a SYBR Green Pro Taq HS kit (Accurate Biology, Cat#AG11701). The primer sequences are listed in Table S2 in the Supporting Information.

### Western Blot Analysis

Cancer cells were washed three times with ice‐cold PBS and lysed with an appropriate volume of radioimmunoprecipitation assay buffer containing protease inhibitors (Solarbio, Cat#R0010). Extraction of cytoplasmic and nuclear proteins was performed using a Nuclear and Cytoplasmic Protein Extraction Kit (Beyotime, Cat#P0028). The sources of the antibodies used are listed in Table S3 in the Supporting Information.

### Tissue Collection and Histological Analysis

Xenograft tumors and lung tissues were fixed, sectioned, and stained according to the manufacturer's instructions (ZSGB‐BIO, Cat#PV‐6000, ZLI‐9017). IHC staining of ATF4 was quantitatively analyzed using Image J, and the average optical density (AOD) was used for statistical analysis. AOD  =  integrated optical density (IOD)/area of positive ATF4 staining in each IHC staining image.

### RNA‐Sequencing (RNA‐seq) Analysis

Raw RNA‐seq data were obtained according to the manufacturer's instructions (BGI). GSEA was used to identify the pathways in which genes with the most significant changes in expression were concentrated in the selected samples. The analysis of these pathways was performed using an algorithm developed by Broad Institute.^[^ ^57^ ^]^

### Survival Analysis for Cancer Patients

The pan‐cancer prognostics database PROGgeneV2 was used to study the prognostic implications of genes in 21 cancer types.^[^ ^58^ ^]^ Relapse‐free survival, metastasis‐free survival, and overall survival among patients in different groups were calculated using the Kaplan–Meier method, and the log‐rank *p*‐value was also computed. Prognostic variables were examined using a Cox proportional hazards model.^[^ ^59^ ^]^

### Statistical Analysis

All statistical analyses were performed using the GraphPad Prism software (version 9.0). The results of the assays are shown as mean ± standard error of the mean (SEM). Statistics of the two sets of data were assessed using Student's two‐tailed *t*‐test. Statistical analyses of tumor growth were performed using a two‐way analysis of variance (ANOVA). Log‐rank tests were used for survival analyses. *p* < 0.05 was considered as statistically significant.

## Conflict of Interest

The authors declare no conflict of interest.

## Author Contributions

Conception and design: X.L. and C.L.; development of methodology: C.Z., P.L., X.L., J.Y., G.Z.; acquisition of data: C.Z., P.L., X.L., Y.Z.; writing, review, and/or revision of the manuscript: X.L., C.Z., L.H.; study supervision: X.L.

## Supporting information

Supporting Information

## Data Availability

The data that support the findings of this study are available from the corresponding author upon reasonable request.
