# Supplementary material for: Near‐Death Cells Cause Chemotherapy‐Induced Metastasis via ATF4‐Mediated NF‐κB Signaling Activation
Source: Adv Sci (Weinh). 2023 Feb 5;10(10):2205835. doi: 10.1002/advs.202205835 (PMC10074103; doi:10.1002/advs.202205835)
Supplement: Supplementary file 1 — Supporting Information [file ADVS-10-2205835-s001.pdf]

ADVANCED SCIENCE
Open Access

Supporting Information

for *Adv. Sci.*, DOI 10.1002/advs.202205835

Near-Death Cells Cause Chemotherapy-Induced Metastasis via ATF4-Mediated NF-κB Signaling Activation

*Chenchen Zhu, Pei Liu, Chuan-Yuan Li, Yuli Zhang, Jiang Yin, Linlin Hou, Guopei Zheng and Xinjian Liu**

Supporting Information

Title: Near-death cells cause chemotherapy-induced metastasis via ATF4-mediated NF-κB signaling activation

Authors: Chenchen Zhu, Pei Liu, Chuan-Yuan Li, Yuli Zhang, Jiang Yin, Linlin Hou, Guopei Zheng, Xinjian Liu*

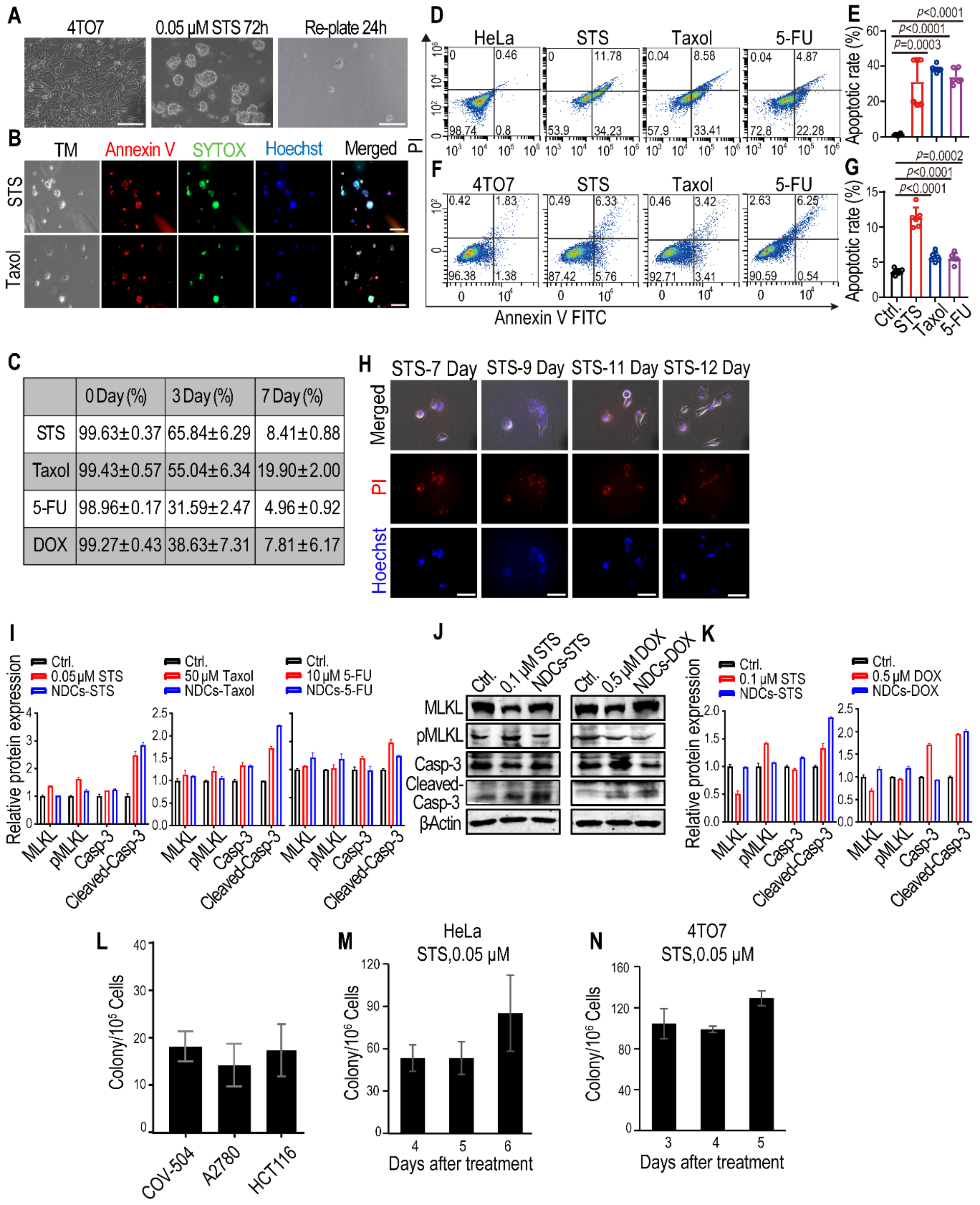

	0 Day (%)	3 Day (%)	7 Day (%)
STS	99.63±0.37	65.84±6.29	8.41±0.88
Taxol	99.43±0.57	55.04±6.34	19.90±2.00
5-FU	98.96±0.17	31.59±2.47	4.96±0.92
DOX	99.27±0.43	38.63±7.31	7.81±6.17

Figure S1. Cytotoxin-induced near-death cancer cells. A) Representative images of normal cultured parental 4TO7 cells (left panel), floating 4TO7 cells after 0.05×10^{-6} M STS treatment for 72 h (middle panel), and the status of adherent of floating STS-treated 4TO7 cells for 24 h (right panel). The scale bars represent 200 μm. B) Annexin V-PE/SYTOX-Green/Hoechst staining showing compromised plasma membrane of adherent cells derived from 0.05×10^{-6} M STS- (upper panel) and 50×10^{-6} M Taxol-induced (lower panel) floating 4TO7 cells. TM presents transmission light images. The scale bars represent 100 μm. C) The percentage of SYTOX-Green positive in all re-adherent cells. The statistical data of Figure 1D-G. Data are presented as the mean ± SEM, $n = 3$. D-G) Apoptosis analyses of repopulated cancer cells (Day 30) from cytotoxin-induced floating HeLa cells (D,E) and 4TO7 cells (F,G) by flow cytometry. High percentage of apoptotic cells existed in cytotoxin-treated

repopulation cancer cells even after 30 days culture in comparison with that of parental cancer cells. Data are presented as mean ± SEM. $n = 3$ biological replicates. p values were determined by Student's t-test. H) 4TO7 cells were treated with STS at dose of 0.05 x 10^{-6} M for 4 days, then the floating cells were plated on 6-well plate with fresh medium. Attached cells were stained with PI (0.5 mg ml^{-1}) and Hoechst 33258 (10 mg ml^{-1}) on day 1 after replating, then the cell division was tracked on day 7, 9, 11 and 12. The scale bars represent 100 µm. I) The quantitative analyses of Figure 1I. J) Western blot images and K) quantification of the expression of apoptosis (cleaved caspase-3, total caspase-3), necroptosis (phosphor-MLKL, pMLKL) markers in cytotoxin-treated cells (harvested on 72 h after treatment) and cytotoxin-derived HeLa NDCs. *β*-Actin was used as the protein loading control. L) Crystal violet staining analyses the number of colonies developed from 1×10^5 floating dying COV-504, A2780 ovarian cancer cells and HCT116 colorectal cancer cells after STS treatment (0.2 x 10^{-6} M for COV-504, and A2780, 0.05 x 10^{-6} M for HCT116). Data are presented as mean ± SD. $n = 2$ biological replicates. M-N) Crystal violet staining analyses the number of colonies developed from 1×10^6 floating dying HeLa cells (M), and 4TO7 cells (N), which collected and replated on indicated time after treatment with 0.05 x 10^{-6} M STS. Data are presented as mean ± SEM. $n = 3$ biological replicates.

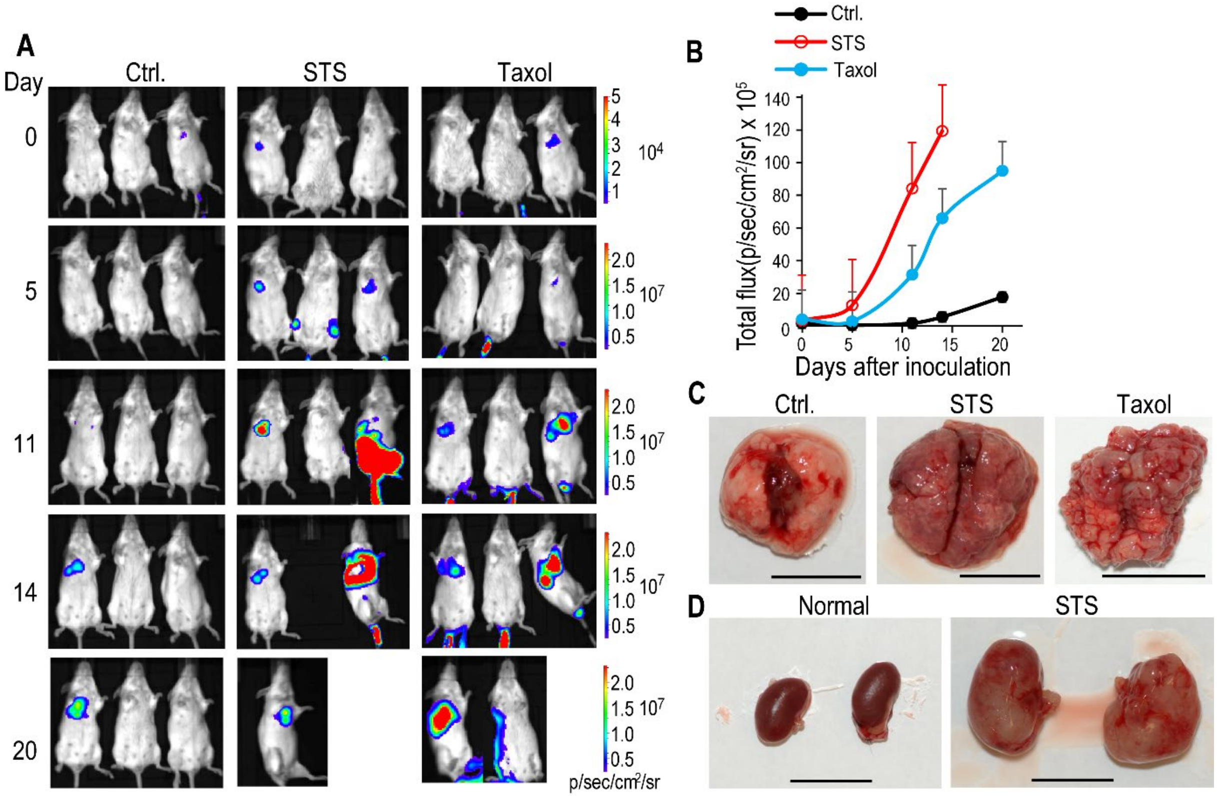

Figure S2. Floating dying cells gain the metastasis ability. A) Representative bioluminescent images of BALB/c mice with intravenous inoculation 2×10^5 4TO7-Luc cells (Ctrl.) or floating dying cells without recovery that collected on day 5 after 0.05 x 10^{-6} M STS or 50 x 10^{-6} M Taxol treatment. B) The dynamic quantitative analysis of the bioluminescence intensity in Figure S2A. Data are presented as mean ± SEM. n = 3 mice per group. C) Representative gross photography showing apparent pulmonary metastases in lungs of mice that intravenous inoculation with STS- and Taxol-treated 4TO7-Luc cells. D) Gross photography showing the kidney metastasis after 11 days of intravenous inoculation floating cells derived from STS-treated 4TO7-Luc cells (right panel). Left panel shows the kidneys of normal BALB/c mouse. The scale bars in (C) and (D) present 1 cm.

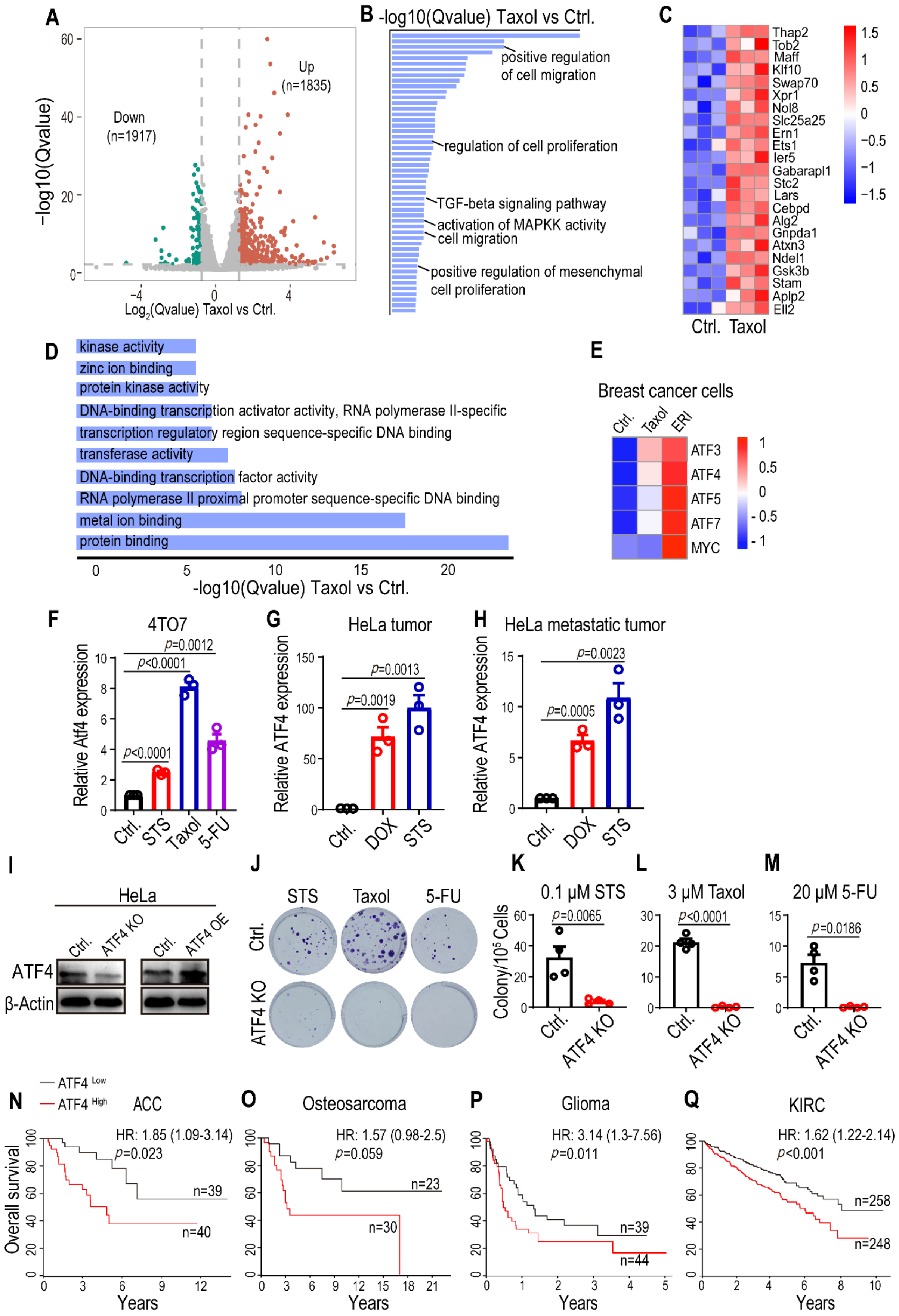
A
Up
(n=1835)
Down
(n=1917)
-log10(Qvalue)
Log2(Qvalue) Taxol vs Ctrl.
B -log10(Qvalue) Taxol vs Ctrl.
positive regulation of cell migration
regulation of cell proliferation
TGF-beta signaling pathway
activation of MAPKK activity
cell migration
positive regulation of mesenchymal cell proliferation
C
Thap2
Tob2
Maff
Klf10
Swap70
Xpr1
Nol8
Slc25a25
Ern1
Ets1
Ier5
Gabarapl1
Stc2
Lars
Cebpd
Alg2
Gnpda1
Atxn3
Ndel1
Gsk3b
Stam
Aplp2
Ell2
Ctrl. Taxol
D
kinase activity
zinc ion binding
protein kinase activity
DNA-binding transcription activator activity, RNA polymerase II-specific
transcription regulatory region sequence-specific DNA binding
transferase activity
DNA-binding transcription factor activity
RNA polymerase II proximal promoter sequence-specific DNA binding
metal ion binding
protein binding
-log10(Qvalue) Taxol vs Ctrl.
E
Breast cancer cells
Ctrl. Taxol ERI
ATF3
ATF4
ATF5
ATF7
MYC
F 4TO7
Relative Atf4 expression
p=0.0012
p<0.0001
p<0.0001
Ctrl. STS Taxol 5-FU
G HeLa tumor
Relative ATF4 expression
p=0.0013
p=0.0019
Ctrl. DOX STS
H HeLa metastatic tumor
Relative ATF4 expression
p=0.0023
p=0.0005
Ctrl. DOX STS
I HeLa
Ctrl. ATF4 KO Ctrl. ATF4 OE
ATF4
β-Actin
J STS Taxol 5-FU
Ctrl.
ATF4 KO
K 0.1 μM STS
Colony/10^5 Cells
p=0.0065
Ctrl. ATF4 KO
L 3 μM Taxol
p<0.0001
Ctrl. ATF4 KO
M 20 μM 5-FU
p=0.0186
Ctrl. ATF4 KO
N ACC
ATF4 Low
ATF4 High
Overall survival
HR: 1.85 (1.09-3.14)
p=0.023
n=39
n=40
Years
O Osteosarcoma
HR: 1.57 (0.98-2.5)
p=0.059
n=23
n=30
Years
P Glioma
HR: 3.14 (1.3-7.56)
p=0.011
n=39
n=44
Years
Q KIRC
HR: 1.62 (1.22-2.14)
p<0.001
n=258
n=248
Years

Figure S3. ATF4 plays an essential role in cancers after chemotherapy. A) Volcano plot analysis of differentially gene expression in 4TO7 Ctrl., and Taxol-induced NDCs. B) GO enrichment analysis of 3752 differential genes that positively enriched in proliferation and migration signal pathways in Taxol-induced 4TO7 NDCs. C) Heatmap analyses showing high expression of 23 invasion-related genes in Taxol-induced 4TO7 NDCs. D) KEGG analysis showing enrichment transcription factor-related signaling pathways in Taxol-induced 4TO7 NDCs. E) Heatmap analyses of 27 breast cancer cell lines showing high expression of activating transcription factor genes and MYC in Taxol- and ERI-treated breast cancer cells. F) RT-PCR analyses the expression of *Atf4* in 4TO7 cells, NDCs derived from STS-, Taxol-, and 5-FU-treated 4TO7 cells. Data are presented as mean ± SEM. $n = 3$. p values were determined by Student's t-test. G,H) RT-PCR analyses the expression of *ATF4* in primary tumor tissues (G) and metastatic tumor tissues (H) derived from cytotoxin-induced HeLa-Luc NDCs ($n = 3$ mice per group). Data are presented as mean ± SEM. p values were determined by Student's t-test. I) ATF4 expression in HeLa cells with an ATF4 knockout (ATF4 KO) (left panel), and ATF4 over-expression (ATF4 OE) (right panel). β-Actin was used as protein loading control. J) Representative crystal violet staining and the numbers of repopulated colonies from 1×10^5 floating HeLa cells or HeLa-ATF4 KO cells that collected and re-plated after K) 0.1 x 10^{-6} M STS, L) 3 x 10^{-6} M Taxol or M) 20 x 10^{-6} M 5-FU treatment for 72 h. Data are presented as mean ± SEM. $n = 4$ biological replicates. p values were determined by Student's t-test. N-Q) High ATF4 expression was strongly associated with worsened outcome in human cancers (ACC, Osteosarcoma, Glioma, KIRC). The patients were arbitrarily classified into ATF4-high and ATF4-low groups. The online tool PROGgene V2 was used, and the Kaplan-Meier curves of survival are shown. For the log-rank test, p values and hazard ratios (HR) are indicated.

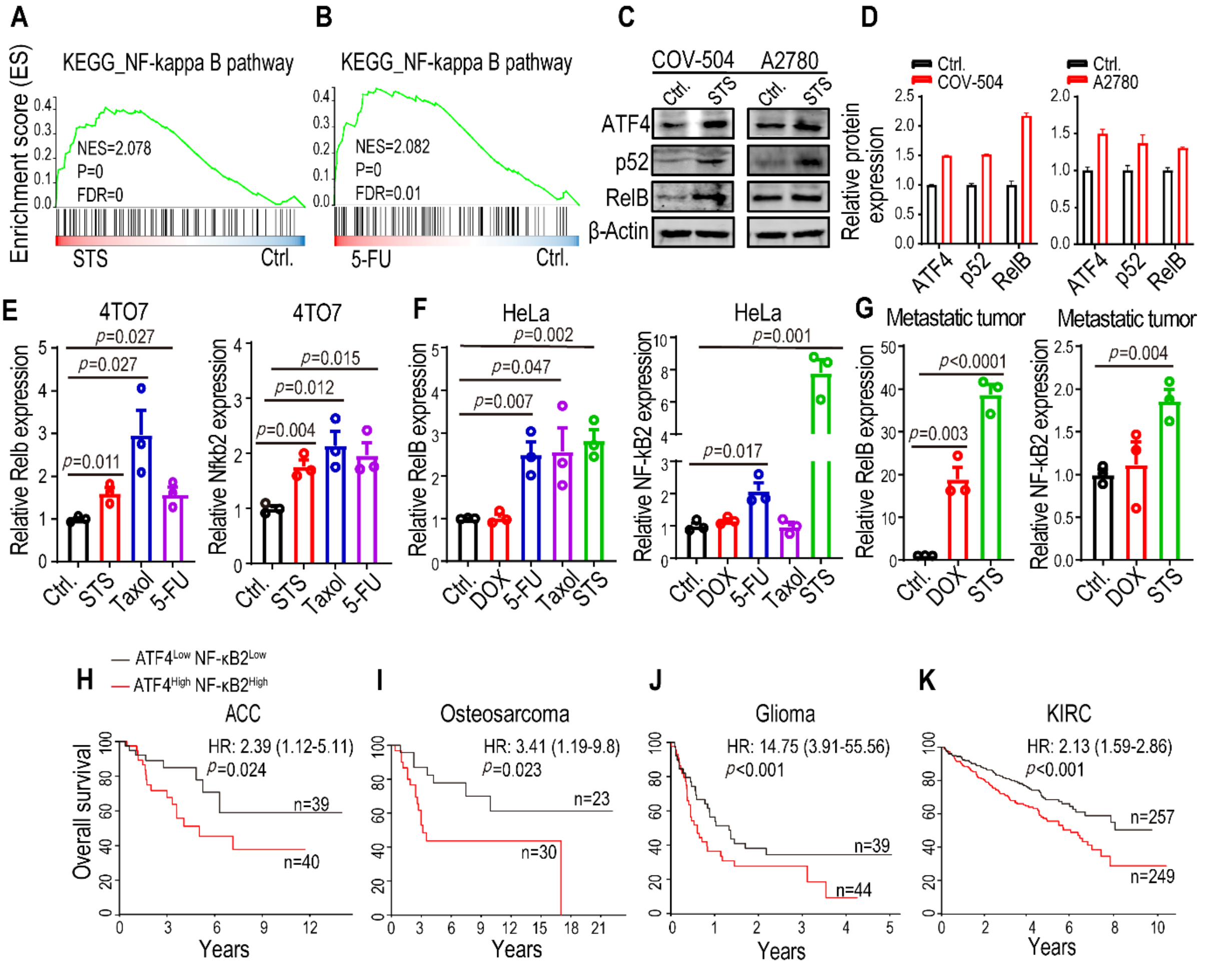

Figure S4. ATF4/NF-*κ*B2 plays a key role in a variety of human cancers. A,B) GSEA analysis showing the significant enrichment of NF-*κ*B signaling pathway in STS- (A), 5-FU-induced NDCs 4TO7 (B) (25 days recovery after 0.05 x 10^{-6} M STS, and 10 x 10^{-6} M 5-FU treatment for 72 h). C) Western blot images and D) quantification of ATF4, p52, and RelB expression in 0.2 x 10^{-6} M STS treatment-derived COV-504 and A2780 NDCs. *β*-Actin was used as protein loading control. E,F) RT-PCR analyses the expression of *RelB* (left panel) and *NF-κB2* (right panel) in control cells, cytotoxin-induced 4TO7 NDCs (E) and HeLa NDCs (F). Data are presented as mean ± SEM. $n = 3$. *p* values were determined by Student's t-test. G) RT-PCR analyses the expression of *RelB* (left panel) and *NF-κB2* (right panel) in metastatic tumor tissues from cytotoxin-induced NDCs HeLa-Luc. ($n = 3$ tumor per group). Data are presented as mean ± SEM. *p* values were determined by Student's t-test. H-K) High co-expression of ATF4/NF-*κ*B2 was strongly associated with worsened outcome in human cancers (ACC, Osteosarcoma, Glioma, KIRC). The patients were arbitrarily classified into ATF4-high/NF-*κ*B2-high and ATF4-low/NF-*κ*B2-low group. The online tool PROGgene V2 was used, and the Kaplan-Meier curves of survival are shown. For the log-rank test, *p* values and hazard ratios (HR) are indicated.

Supplementary Methods

Cells culture conditions

HeLa human cervical cancer cells were obtained from Cell Culture Facility of Chinese Academy of Sciences. Its identity was verified by the STR method. 4TO7 mouse breast cancer cell line, highly tumorigenic but non-metastatic, was a kind gift from Dr. Mark W. Dewhirst from Duke University. Human ovarian carcinoma cells COV-504, and A2780 were obtained from Guangzhou Cellcook Biotech Co. Ltd. Human colorectal carcinoma cells HCT116, 293T cells were purchased from ATCC (Manassas, VA). 4TO7, HeLa cells were grown in RPMI-1640 (Gibico, Thermo Scientific) with 10% fetal bovine serum (FBS), penicillin (100 units ml^{-1}), and streptomycin (100 μg ml^{-1}). HCT116, 293T, COV-504 and A2780 cells were grown in DMEM (Gibico, Thermo Scientific) containing 10% fetal bovine serum (FBS, Gibico, Thermo Scientific), penicillin (100 units ml^{-1}), and streptomycin (100 μg ml^{-1}). All cell lines were subjected to mycoplasma test periodically by use of the Universal Mycoplasma Detection Kit (ATCC).

Construction of lentivirus vectors

Knockout cells were generated with lentivirus-mediated CRISPR-Cas9 technology. Single guided RNA (sgRNA) sequences were designed using a public-domain online CRISPR design tool. The oligonucleotides used for sgRNA cloning and recombinant plasmid sequencing were listed in Table S2. Double-stranded oligonucleotides encoding the sgRNA sequences were cloned into BsmBI (Thermo Fisher Scientific) digested plasmid LentiCRISPRv2 (deposited by F. Zhang of MIT to Addgene, Cambridge, MA) which co-expresses Cas9 and sgRNA in a single

vector. The sgRNA-encoding CRISPR lentivirus vectors were then produced using an established protocol from the Trono laboratory (https://www.epfl.ch/labs/tronolab/laboratory-of-virology-and-genetics/ lentivectors-toolbox/). To generate the knockout cell lines, target cells were infected with sgRNA-encoding CRISPR lentivirus and cultured in RPMI-1640 with 10% FBS and selected in hygromycin (200 μg ml^{-1} for HeLa cells, 300 μg ml^{-1} for 4TO7 cells) for 7-10 days. Full-length mouse and human *ATF4* were generated by PCR from cDNA derived from the 4TO7 cells and 293T cells. These fragments were then cloned into the pLEX-MCS lentiviral vector for gene expression. DNA sequence for the HA tag was constructed to the 3' end of *ATF4*. The primers used to construct the recombinant vector and recombinant plasmid sequencing were listed in Table S2.

Plasma membrane integrity and apoptosis analysis

The floating cells after cytotoxin exposure and control cells were labeled with SYTOX-Green nucleic acid (Invitrogen, Cat#S11348) and Annexin V-PE (Beijing 4A Biotech, Cat#FXP027) according to manufacturer's instructions. The processed samples were analyzed by use of CytoFLEX Flow Cytometer.

Mouse models

2×10^5 floating cells without recovery by Taxol- and STS-treatment and 4TO7-Luc cells were intravenously injected into the tail vein of BALB/c mice. Mice were anesthetized with isoflurane and injected (intraperitoneally) with D-luciferin potassium salt (GOLDBIO) at dose of (150 mg kg^{-1}) on days 0, 5, 11, 14 and 20 after tumor cells injection. 4TO7-Luc lung

metastases were assessed by in vivo bioluminescence imaging using IVIS Spectrum. The integrated light intensity measured by single photon counting with 10-minute exposure was used to quantify the amount of light emitted by 4TO7-Luc cells. A low-intensity visible light image was made for overlay images.

Table S1. Key resources used in this study

Reagent or Resource	Source	Identifier
Peroxidase Blockers	ZSGB-BIO	Cat# PV-6000
DAB	ZSGB-BIO	Cat#ZLI-9017
TRIzol™ reagent	Invitrogen	Cat#44894
Isoflurane	RWD	Cat#R510-22-10
Staurosporine	Cell Signaling Technology	Cat#9953S
Paclitaxel	MedChemExpress (MCE)	Cat#HY-B0015
5-fluorouracil	MedChemExpress (MCE)	Cat#HY-90006
Doxorubicin	Selleckchem	Cat#S1208
SYTOX-Green	Invitrogen	Cat#S11348
Annexin V-PE	Beijing 4A Biotech	Cat#FXP027
Hoechst 33258	Sigma-Aldrich	Cat#B1155
RPMI-1640	Gibico	Cat#31800022
DMEM	Gibico	Cat#12800017
Trypsin-EDTA	ThermoFisher	Cat#25200072
Fetal Bovine Serum	Gibico	Cat#10100-147
BsmBI	ThermoFisher	Cat#FD0454
D-luciferin potassium salt	GOLDBIO	Cat#MB102
D-luciferin potassium salt	Beyotime	Cat#ST196
Annexin V-FITC Apoptosis kit	MultiSciences	Cat#AP101
Nuclear and Cytoplasmic Protein Extraction Kit	Beyotime	Cat#P0028
Superscript II reverse transcriptase	Invitrogen	Cat#18064-014
SYBR Green Pro Taq HS kit	Accurate Biology	Cat#AG11701
BCA Protein Assay Kit	Beyotime	Cat#P0012
Universal Mycoplasma Detection Kit	ATCC	N/A
CRISPR design tool	https://chopchop.cbu.uib.no	N/A
PROGgeneV2	http://www.progtools.net/gene/index.php	N/A
CytoFLEX Flow Cytometer	Beckman Coulter, Inc.	N/A
GraphPad Prism 9	https://www.graphpad.com/scientific-software/prism/	N/A
ImageJ	https://imagej.nih.gov/ij/	N/A

Table S2. Primers and oligos used in this study

Name	Sequence (5'-3')	Purpose
Human ATF4 sgRNA-F	CACCGTTTGATAGAAGAGGTCCGCA	Cloning of human ATF4 sgRNA
Human ATF4 sgRNA-R	AAACTGCGGACCTCTTCTATCAAAC	Cloning of human ATF4 sgRNA
Mouse Atf4 sgRNA-F	CACCGAGATGAGCTCTTGACCACGT	Cloning of mouse Atf4 sgRNA
Mouse Atf4 sgRNA-R	AAACACGTGGTCAAGAGCTCATCTC	Cloning of mouse Atf4 sgRNA
Seq-plenti-1718	AGGGCCTATTTCCCATGATTC	Recombinant plasmid for sequencing
SpeI-hATF4-F	GACTAGTGCCACCATGACCGAAATGAGCTTCCTG	Cloning of human ATF4 for expressing
SpeI-hATF4-R	ATAGTTTAGCGGCCGCGGGGACCCTTTTCTTCCCC	Cloning of human ATF4 for expressing
SpeI-mAtf4-F	GACTAGTCCATGACCGAGATGAGCTTCCTGAA	Cloning of mouse Atf4 for expressing
SpeI-mAtf4-R	ATAAGAATGCGGCCGCCGGAACTCTCTTCTTCCCCCTTG	Cloning of mouse Atf4 for expressing
CMV-F	CACCAAAATCAAGGGACTT	Recombinant plasmid for sequencing
mouse Actb-F	GTGACGTTGACATCCGTAAAGA	Real-time quantitative PCR
mouse Actb-R	GCCGGACTCATCGTACTCC	Real-time quantitative PCR
human ACTB-F	CATGTACGTTGCTATCCAGGC	Real-time quantitative PCR
human ACTB-R	CTCCTTAATGTCACGCACGAT	Real-time quantitative PCR
mouse Atf4-F	CCTGAACAGCGAAGTGTTGG	Real-time quantitative PCR
mouse Atf4-R	TGGAGAACCCATGAGGTTTCAA	Real-time quantitative PCR
human ATF4-F	ATGACCGAAATGAGCTTCCTG	Real-time quantitative PCR
human ATF4-R	GCTGGAGAACCCATGAGGT	Real-time quantitative PCR
mouse Relb-F	CCAAAGCCGTTCTCCTTAATGTA	Real-time quantitative PCR
mouse Relb-R	GTTCCAGTGACCTCTCTTCCC	Real-time quantitative PCR
human RelB-F	CAGCCTCGTGGGGAAAGAC	Real-time quantitative PCR
human RelB-R	GCCCAGGTTGTTAAAACTGTGC	Real-time quantitative PCR
mouse Nfkb2-F	TGGCATCCCCGAATATGATGA	Real-time quantitative PCR
mouse Nfkb2-R	TGACAGTAGGATAGGTCTTCCG	Real-time quantitative PCR
human NFKB2-F	ATGGAGAGTTGCTACAACCCA	Real-time quantitative PCR
human NFKB2-R	CTGTTCCACGATCACCAGGTA	Real-time quantitative PCR

Forward: F, Reverse: R, Human: h, Mouse: m

Table S3. Antibodies used in this study

Antibodies	Source	Identifier	Dilution
rabbit anti-ATF4	Proteintech	Cat#10835-1-AP; RRID:AB_2058600	1:1000 for WB; 1:300 for IHC
mouse anti-Luciferase	Abbkine	Cat#ABM40300	1:200 for IHC
rabbit anti-MYC	Proteintech	Cat#10828-1-AP; RRID:AB_2148585	1:1000 for WB
rabbit anti-GAPDH	Proteintech	Cat#60004-1-Ig; RRID:AB_2107436	1:20000 for WB
mouse anti-β-Actin	Proteintech	Cat#66009-1-Ig; RRID:AB_2687938	1:5000 for WB
mouse anti-PCNA	Proteintech	Cat#60097-1-Ig; RRID:AB_2236728	1:1000 for WB
rabbit anti-NIK	Cell Signaling Technology	Cat#4994; RRID:AB_2297422	1:1000 for WB
rabbit anti-Phospho-IKKα/β (Ser176/180)	Cell Signaling Technology	Cat#2697; RRID:AB_2079382	1:1000 for WB
rabbit anti-Phospho-NF-κB2 p100 (Ser866/870)	Cell Signaling Technology	Cat#4810; RRID:AB_659925	1:1000 for WB
rabbit anti-NF-κB2 p100/p52	Cell Signaling Technology	Cat#4882; RRID:AB_10695537	1:1000 for WB
rabbit anti-RelB (C1E4)	Cell Signaling Technology	Cat#492; RRID:AB_2179173	1:1000 for WB
rabbit anti-Caspase 3	Cell Signaling Technology	Cat# 9665; AB_2069872	1:1000 for WB
rabbit anti-Cleaved Caspase 3	Abcam	Cat# 9661; RRID:AB_2341188	1:1000 for WB
rabbit anti-MLKL	Abcam	Cat# ab184718; RRID:AB_2755030	1:1000 for WB
rabbit anti-MLKL (phospho S345)	Abcam	Cat# ab196436; RRID:AB_2687465	1:1000 for WB

WB: Western blot, IHC: Immunohistochemistry
